# The interaction of Cations with Solutes in an Aqueous Solution

**DOI:** 10.1021/acs.jpcb.5c02947

**Published:** 2025-07-11

**Authors:** Akshay Malik, Arun Yethiraj

**Affiliations:** Department of Chemistry, University of Wisconsin-Madison, Madison, Wisconsin 53706, United States

## Abstract

Lithium ion batteries are important in a number of applications, and the demand for Lithium is expected to increase. In typical industrial Li^+^ extraction, brine is pumped to the surface of large reservoirs, followed by chemical treatment. This method requires large quantities of water and can pollute groundwater. There has been considerable interest in developing membranes that can be used for preferential Lithium recovery. It has been suggested that para-toluene sulfonate (PTS) can preferentially bind Li^+^ over other cations. In this work, we study the physical chemistry of Li^+^, Na^+^, and K^+^ binding to solutes in an aqueous solution via combined classical and ab initio molecular dynamics simulations. The solutes we consider are PTS, a trimer of PTS, and 4-(trifluoromethyl)­benzenesulfonate (TBS). From the analysis of radial distribution functions and the potential of mean force (PMF), we find that the sulfonate group in the cations has the strongest spatial correlation with Li^+^ followed by Na^+^ and then K^+^. The magnitude of PMF, however, is only of the order of the thermal energy. The correlation between the sulfonate group and Li^+^ can be enhanced by polymerizing the PTS molecule, providing a possible means of extracting Li^+^ from the aqueous phase. For PTS, K^+^ is more strongly correlated with the carbon atoms in the benzene ring than the sulfonate group. This might provide a resolution to the puzzling difference between the behavior of sulfonated polystyrene polymers in the presence of K^+^ and Li^+^ counterions.

## Graphical abstract

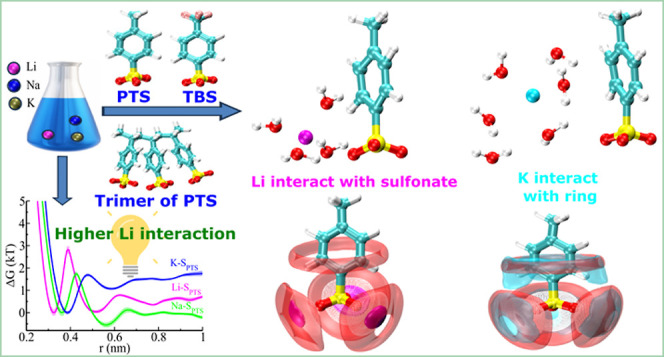

## 1 Introduction

Lithium is an invaluable resource used in rechargeable batteries for a variety of applications. The global demand of Lithium (Li) has grown significantly over the last two decades and is expected to grow even more significantly in the future.^1^ In 2015, the demand of Li in Lithium carbonate equivalent was 263,000 t, and this is expected to increase to about 2 million t by 2030. One of the driving forces for Lithium demand is the burgeoning use of Li-ion batteries in electric vehicles (EVs) and plug-in hybrid electric vehicles.^2^ A large-scale switch away from internal combustion engines is expected, driven by concerns about climate change due to global greenhouse gas emissions, in addition to health concerns from pollutants. This could push Lithium demand beyond current projections, thus making enhanced production crucial. Lithium exists in land sources as ores or in brines, and in seawater.^3^ Direct extraction from any one of these sources is time-consuming. Polymeric membranes have been suggested as a means of direct liquid extraction using selective ligands.^2,4–11^ In this work, we investigate the interaction of cations with solutes using a multiscale computational approach.

The main sources of Lithium are in igneous rocks, sedimentary rocks, and brine solutions.^1,3–5,7^ Recovery of Lithium from rock ores and clay is usually done using hydro- and pyro-metallurgy. The first step is the beneficiation of the mineral using a variety of methods including magnetic separation, gravity concentration, electrostatic separation, and flotation.^1,3^ The concentrated ore is then either calcined or roasted followed by leaching to dissolve the Lithium into an aqueous phase. Li_2_CO_3_ is produced through carbonation and then precipitated. Lithium also occurs in significant amounts in seawater, geothermal waters, and brines, which contain roughly 60% of the total Lithium resources.^7^ Existing methods for Lithium extraction from brine include evaporation, direct precipitation, membrane-related processes, solvent extraction, sorption, and ion exchange.

The most widely used method for Lithium extraction from brine is evaporation.^5^ The brine is pumped into large evaporation ponds, where it remains for months, or even years, until the water has evaporated. During the evaporation process, less soluble salts precipitate first, and the Lithium concentration increases to about 6% by weight. Further processing is required to remove ions such as Mg^2+^ and Ca^2+^, and the Lithium recovery is about 50–80%. The process is very time-consuming, and the ponds must be exposed to direct sunlight. There are also significant environmental concerns. The process requires significant amounts of freshwater; an estimate is that one ton of Lithium metal requires 500,000 gallons of water.^12^

Polymeric membranes can be an attractive method for brine purification.^2,4–11^ They are easily scalable, are energy efficient, and are widely used for water purification. A key challenge in the extraction of Lithium from brine is the low Li^+^ concentration (0.3–1.5%) and the presence of other ions, e.g., Na^+^, K^+^, Mg^2+^, and Ca^2+^, with similar physical properties, and at higher concentrations. Selectively extracting Li^+^ from a solution containing K^+^ is challenging.

An increase in the demand for EVs has led to the generation of many spent lithium ion batteries (LIBs), thus causing significant environmental pollution and health issues due to their toxic contents,^13^ Recycling strategies for properly treating spent LIBs are therefore important.^14^ Recently, *p*-toluene sulfonic acid (PTSA) emerged as a potential candidate for Li recovery from spent LIBs.^15,16^ Yadav et al. employed PTSA for leaching LIBs and found high Li and iron recovery yields from spent Li-iron phosphate batteries.^15^ Furthermore, cathode materials for LIBs could be prepared from the extracted metals. Liu et al. utilized PTSA to recycle Li and cobalt from wasted LIBs and synthesized raw battery materials from the extracted metals.^16^ Sreejith and Sen employed Li and para-toluene sulfonate (PTS) salt in LIBs.^17^ The Li-PTS salt is stable at high temperatures and has high conductivity at room temperature, making it suitable for applications in LIBs at high temperatures.^17^

Several experimental studies suggest that PTS can be potentially exploited for Li extraction.^15–17^ However, fundamental studies focusing on the interactions of Li with PTS are scarce in the literature. In this work, we investigate the possible selective extraction of Li^+^ from a mixture of Li^+^, Na^+^, and K^+^ in water using PTS. We also study two PTS variants to increase extraction efficiency and Li^+^ selectivity. The first is the trimer of PTS, which strives to elucidate the effect of oligomers on the local structures during the extraction process. The second is fluorinating the methyl hydrogens in PTS, leading to the formation of 4-(trifluoromethyl)­benzenesulfonate (TBS) to investigate the effect on cation selectivity by changing the side chain of PTS with the electron-withdrawing group.

The interaction of cations with polyions is of interest from a fundamental standpoint. Muthukumar^18^ has proposed that the dipoles formed from the binding of cations to polyions is the key effect in the so-called “ordinary to extraordinary” transition observed in dynamic light scattering measurements of polymers, where the diffusion constant of the polyion increases sharply, with increasing concentration, from the Stokes–Einstein value at low concentrations. There is also an interesting cation effect with differences in properties with Na^+^ or K^+^ cations. For example, the solubility of the polymer poly­[di- (carboxylatophenoxy)­phosphazene] (PCPP) is much higher in KCl compared to NaCl solutions, where coacervates are formed.^19^ In studies of coacervate formation with PSS, the salt of choice is generally KBr,^20–22^ which is thought to lead to stronger interactions between the ions and polymer and thus disrupt complexes. In contrast, most theoretical work treats Na^+^ and K^+^ as generic monovalent cations.

In this work, we study the interaction and correlation of cations with PTS and PSS using molecular simulations. Using multiscale simulation studies, including gas-phase calculations, classical molecular dynamics (CMD) simulations, and ab initio molecular dynamics (AIMD) with enhanced sampling techniques, we study the cation binding sites in PTS/TBS and the role of water in the extraction process. We compare CMD and AIMD simulations to determine if quantum effects are needed to explain the physics of cation extraction using PTS/TBS in the aqueous phase or if CMD is sufficient. We draw two main conclusions. The first is that the “binding” of cations to the charged groups is weak; i.e., the free energy of association is of the order of the thermal energy and insufficient to create a dipole with significant lifetime. The second is that there is a significant difference between K^+^ and Li^+^ with the former binding to the aromatic ring and the latter to the charged sulfonate group.

## 2 Simulation Details

There are several simulation methods used in this work. These include gas-phase simulations using classical force fields and density functional theory (DFT), liquid-phase simulations using CMD and AIMD, and enhanced sampling methods using OPES (On-the-fly Probability Enhanced Sampling) with CMD and AIMD. Details of these methods are presented in this section.

One molecule of each cation and PTS/TBS is used in gas-phase calculations. For the aqueous phase, in most cases, the system consists of 64 water molecules, one molecule of the solute (PTS, TBS, or a trimer), and a single cation. For comparison, we also perform simulations with all the three, i.e., Li^+^, Na^+^, and K^+^, cations with Cl^–^ ions added to maintain electroneutrality. Equilibrated configurations showing all simulated systems that are overall electrically neutral are depicted in the Supporting Information (Figure S1).

The CMD simulations are performed using the GROMACS-2019.6 ^23–26^ package with the CHARMM^27–30^ force-field along with the SPC/E water model.^31^ The simulation cell is a cube with periodic boundary conditions in all directions, and initial configurations are created using the PACKMOL^32^ package followed by energy minimization using the steepest descent algorithm and 10 ns of equilibration in the isothermal–isobaric (*NPT*) ensemble (*N* is the number of molecules). The Nosé–Hoover^33,34^ thermostat and Parrinello–Rahman^35^ barostat are used, respectively, to maintain the temperature at 298 K and the pressure at 1 bar.

Bonds involving hydrogen atoms are constrained using the LINCS algorithm,^36^ electrostatic interactions are treated using the particle-mesh-Ewald (PME)^37,38^ method, and short-ranged interactions use a switching function range of 0.5–0.6 nm and a cutoff radius of 0.6 nm. The leapfrog algorithm having a 1 fs time-step is used to integrate the equations of motion. Properties are average over a production run of 50 ns in the *NPT* ensemble (298 K, 1 bar).

AIMD simulations are performed using the CP2K package.^39^ The hybrid Gaussian-plane wave technique in the CP2K Quickstep module^39^ is used to solve the Kohn–Sham DFT equations and calculate the forces and energies of each atom. The Triple-ζ, double polarization basis set^40^ with the Becke-Lee–Yang–Parr (BLYP) functional^41,42^ and the Goedecker–Teter–Hutter (GTH) pseudopotentials^43,44^ are used, with a third-generation D3 dispersion correction^45^ and a 400 Ry plane wave cutoff. Periodic boundary conditions are used in all three dimensions. The system is propagated with a time-step of 0.5 fs in the *NVT* ensemble with a Nosé–Hoover thermostat.^33,34^ Initial configurations of the systems are obtained from equilibrated classical *NPT* simulations and relaxed using geometry optimization. Properties are averaged over production runs of 115 ps.

The potential of mean force (PMF) is an important thermodynamic measure of ion-pairing,^46^ especially when solvent effects are significant.^47^ We employ on-the-fly probability enhanced sampling (OPES)^48^ in both CMD and AIMD simulations to calculate the PMF.

Classical OPES-CMD simulations employ GROMACS-2019.6 ^23–26^ patched with the PLUMED^49–51^ package. We used two collective variables (CVs): The distance *r* between the cation and the Sulfur atom of the solute and the coordination number (CN) of water in the first solvation shell of cations. The choice of these two CVs is based on previous studies of similar systems, which have shown the importance of both *r* and CN in determining the ion pairing in water.^47^ Starting from previously equilibrated systems, 15 ns OPES-CMD simulations in the *NVT* (*V* is the volume) ensemble are performed by applying a history-dependent bias along the CVs at every step.^48^ The reweighting approach^52^ estimates the unbiased distribution and obtains unbiased free energy, whose methodology is taken from a previous work.^53^ For faster free energy convergence, we restrict the sampling of distances *r* by applying a large bias using harmonic lower (*r* ≤ 0.30 nm) and upper (*r* ≥ 0.65 nm) walls similar to the previous work.^53^ These choices are based on the pair correlation functions obtained from CMD simulations which show that the first solvation shell has a range of approximately 0.65 nm. Previous studies have shown that it is crucial to understand the behavior of PMFs at larger distances.^54^ Therefore, we also simulated a larger system with 1024 water molecules without distance restriction to verify that the PMF is flat at a longer distance for a quantitative understanding of the binding free energy.

To compute the statistical uncertainty, we partitioned the production trajectories into three blocks and provided the associated standard deviations in the calculations of radial distribution functions (RDFs) and PMFs.

OPES-AIMD simulations are performed by integrating PLUMED^49–51^ with the CP2K package.^55^ Other details are the same as those in the OPES-CMD simulations.

## 3 Results and Discussion

### 3.1 Gas Phase

In the gas phase, in all cases, the cations bind to the sulfonate group (Figure S2 in the Supporting Information). In the AIMD structures, Li^+^ being the smallest in size is symmetrically positioned between the two oxygens of the sulfonate group, but Na^+^ and K^+^ equidistant from the three sulfonate oxygens, with Na^+^ slightly closer due to being smaller than K^+^. The formal charges on PTS/TBS and cations are −1 and +1, respectively, in the case of CMD simulations. However, total restrained electrostatic potential^56^ charges obtained from the geometry-optimized structures in AIMD simulations fluctuate around −0.8*e* and +0.8*e* for PTS/TBS and cations, respectively, suggesting charge transfer between cations and PTS/TBS. There is no significant difference between PTS and TBS regarding the binding of the cations. The gas-phase structures obtained from CMD are in close agreement with the AIMD results for Na^+^ and K^+^, suggesting that the CHARMM classical force field is accurate for PTS/TBS and these cations.

### 3.2 Liquid Structure

#### 3.2.1 Correlations of Cations with the Solute

Li^+^ shows a strong RDF peak with the oxygen atom of the sulfonate group. This correlation is stronger for PTS than TBS, and it is strongest for the trimer of PTS. These trends are weaker for the Na^+^ and K^+^ cations. Figure 1a–c depicts the RDFs between the cations and the oxygen atom of the sulfonate group in PTS (O_PTS_), trimer of PTS, and TBS, respectively, obtained from CMD simulations. The position of the peak is the same for all of the anions and is a measure of the size of the cation. The nearest neighbor peak for Li^+^ is at 0.19 nm, followed by Na^+^ at 0.23 nm and K^+^ at 0.26 nm. The peak in the Li^+^ RDF has a lower value in the case of TBS. An examination of the force field parameters shows that this is not due to changes in the charge of atoms in the sulfonate group, which are the same for PTS and TBS, but might be attributed to subtle changes in the partial charge distribution in the ring carbon atoms. The higher peak value for the trimer is a cooperative effect of the higher charge density of the anion in this case.

**Figure 1:**
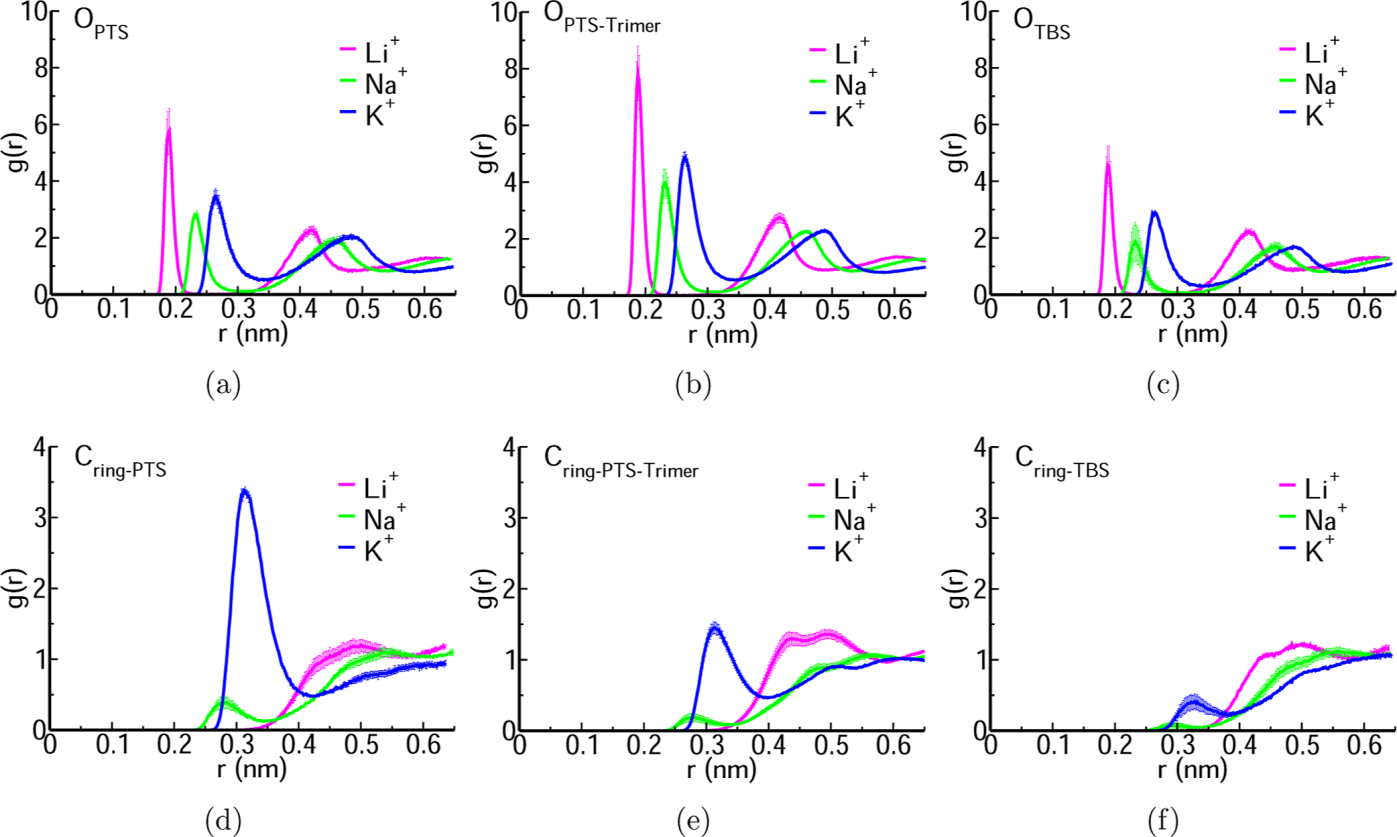
RDFs of Li^+^, Na^+^, and K^+^ with oxygen atoms of sulfonate in (a) PTS (O_PTS_), (b) trimer of PTS (O_PTS–Trimer_), (c) TBS (O_TBS_), and carbon atoms of the benzene ring in (d) PTS (C_ring–PTS_), (e) trimer of PTS (C_ring–PTS–Trimer_), and (f) TBS (C_ring–TBS_).

Figure S3a–c shows a closer association of Li^+^ with the sulfur atom of the O_PTS_, S_PTS‑Trimer_, and S_TBS_, when compared to Na^+^ and K^+^, with peaks positioned at 0.33 nm (Li^+^), 0.37 nm (Na^+^), and 0.39 nm (K^+^). From Figures 1a–c and S3a–c, we conclude that Li^+^ interacts more strongly with the sulfonate group than do Na^+^ and K^+^.

An interesting result is that for PTS, the K^+^ cations are more likely to be closer to the benzene ring than to the sulfonate group. Figure 1d–f depicts the RDFs between the cations and the carbon atoms of the ring in PTS, trimer of PTS, and TBS, respectively. While the correlation between the Li^+^ and Na^+^ cations with the ring carbons is generally weak, there is a strong RDF peak between the K^+^ cation and the ring carbons in the case of PTS, but not the other anions. A consequence of this is that the charge distribution of the anion–cation complex is quite different in the case of K-PTS than in the case of the other anions and cations. Figure S3d–f shows a closer association of K^+^ with carbon atom directly attached to the benzene group in the PTS molecule ( $C_{{CH}_{3}-PTS}$ ) with a peak at 0.43 nm, and this correlation decreases for PTS-Trimer ( $C_{{CH}_{3}-PTS‐Trimer}$ ) and TBS ( $C_{{CF}_{3}-TBS}$ ). The results in Figures 1d–f and S3d–f show that K^+^ has more interaction with the carbon atoms of the benzene ring compared to Li^+^ and Na^+^. This is reminiscent of the cation–pi interactions, also seen in peptides.^57^ This effect is not present in the trimer, possibly because of the steric hindrance from the oligomer backbone.

For understanding effective Li^+^ extraction from aqueous phases, it is crucial to consider systems where Li^+^ coexists with other cations. To investigate this, we consider a system with all three cations and with chloride ions added to preserve electroneutrality. In this case, the correlation between the Li^+^ and the sulfonate group is enhanced, without significantly changing the correlation of the other cations. Figures 2 and S4 depict the RDFs of Li^+^ with the PTS/Trimer of PTS/TBS in the presence of Na^+^, K^+^, and Cl^–^ ions. In a mixture of ions, Li^+^ is found to be in closer association with oxygen and sulfur atoms, highlighting the selective interaction of Li^+^ with the sulfonate group (Figures 2a–c and S4a–c). A higher correlation of the Li^+^–O_PTS‑Trimer_ and Li^+^–S_PTS‑Trimer_ RDF pair suggests that polymerizing the PTS could amplify the Li^+^ interaction with the sulfonate group, thereby facilitating a more effective Li^+^ separation. Thus, the sulfonate group plays a pivotal role in the selective isolation of Li^+^ from an aqueous solution containing mixed ions (Na^+^, K^+^, and Cl^–^).

**Figure 2:**
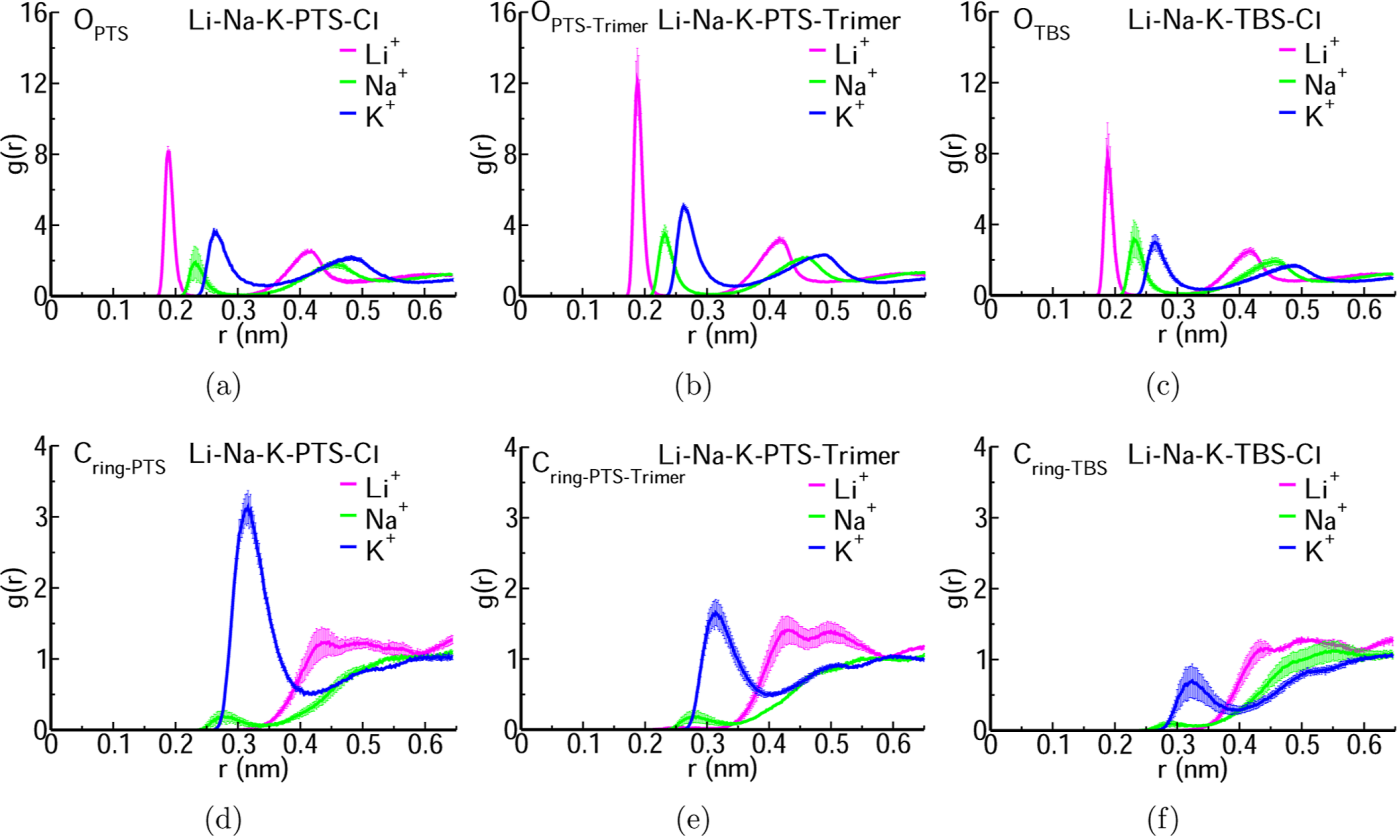
RDFs of Li^+^, Na^+^, and K^+^ with oxygen atoms of sulfonate in (a) PTS (O_PTS_), (b) trimer of PTS (O_PTS–Trimer_), (c) TBS (O_TBS_), and carbon atoms of the benzene ring in (d) PTS (C_ring–PTS_), (e) trimer of PTS (C_ring–PTS–Trimer_), and (f) TBS (C_ring–TBS_) for a system containing Li^+^, Na^+^, and K^+^ ions mixed with PTS, or Trimer of PTS, or TBS and Cl^–^ ions to make the system neutral.

The correlation of Na^+^ and K^+^ is essentially unchanged from that of the single ion case. K^+^ exhibits a stronger RDF peak with C_ring‑PTS_, indicating a preferential interaction of K^+^ with C_ring‑PTS_ in a mixed ion environment (Figures 2d–f and S4d–f). For the purpose of selectively separating Li^+^, the TBS molecule is advantageous due to its reduced interaction of K^+^ with C_ring‑TBS_, in contrast to the K^+^-C_ring‑PTS_ correlation in the PTS molecule. The sulfonate group in the TBS molecule effectively isolates lithium from a mixture of ions, and as a result, the overall selectivity of TBS over PTS for Li^+^ is enhanced. The RDFs presented in Figures 2 and S4 corroborate the observations from Figures 1 and S3, indicating consistent results between mixed systems containing Li^+^, Na^+^, and K^+^ within the same simulation box and those with single cations.

#### 3.2.2 Organization of Water Molecules

There is a stronger RDF peak between the oxygen atom of the water and Li^+^ than the other cations, as expected, because of the stronger field near the surface of the smaller ion. Figures S5–S7 show the radial distributions of the oxygen (O_WAT_) and hydrogen (H_WAT_) atoms of water molecules with the cations and the RDF of H_WAT_ with the atoms of the sulfonate group for PTS, Trimer of PTS, and TBS, respectively. The correlations between the water molecules and ions are independent of the solute. The RDFs are consistent with the O_WAT_ being correlated with the ions and the H_WAT_ atoms pointing away.

The CN of water molecules around the cations is similar to that in aqueous solutions, where the CN (*n*_coord_) is usually 4 for Li^+^ 5 or 6 for Na^+^ with an average of approximately 5.5 and range between 5 and 8 for K^+^ with an average of 6. We find that *n*_coord_ = 3.9, 5.4, and 6.1 for Li^+^ , Na^+^,  and K^+^, respectively.

The organization of the water molecules near the oxygen atom, O_PTS_, of the PTS is consistent with a hydrogen bond. Figure 3 depicts the radial angular distribution function (*g*(*r*, θ)).^58^ The *g*(*r*, θ) plot shows high intensity at a hydrogen-acceptor distance (H_WAT_–O_PTS_) of 0.17 nm and hydrogen-donor–acceptor angle (H–O_WAT_–O_PTS_) of ∼10^°^ which satisfies both the distance and angle geometric criteria for a hydrogen bond^59–61^ where O_PTS_ acts as an acceptor for H_WAT_. These types of hydrogen bonding interactions between sulfonate oxygens and H_WAT_ have been previously reported by Pejov et al. in their quantum chemical study of the PTS–water complex.^62^

**Figure 3:**
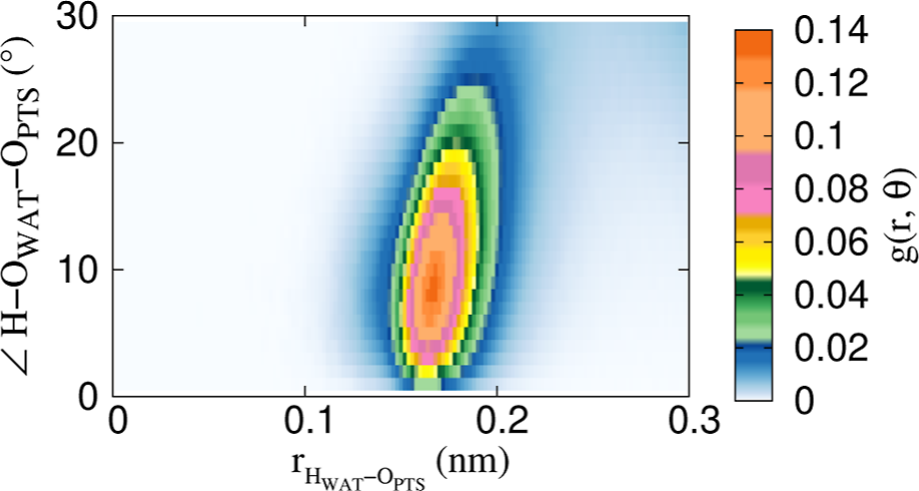
Radial angular distribution function (*g*(*r*, θ)) corresponding to the H_WAT_–O_PTS_ distance and H–O_WAT_–O_PTS_ angle in the Li-PTS system, where H_WAT_ is the hydrogen of water and O_PTS_ refers to the oxygen of the PTS molecule.

#### 3.2.3 Spatial Distribution Functions (SDFs): 3-D Organization

The structure elucidated by the RDFs can be visualized using three-dimensional SDFs. The TRAVIS software is used to calculate the intermolecular SDFs.^63^ PTS/TBS is used as a reference molecule during SDF calculation, with the three reference atoms being two oxygens and a sulfur. The SDF radius cutoff is 0.6 nm. Figure 4 shows the SDFs highlighting the three-dimensional arrangement of cations and water around the reference PTS/TBS molecule. SDFs corresponding to cations are found near the oxygen atoms of sulfonate groups in PTS and TBS. However, circular ring-type isosurfaces highlighting K^+^ in the K-PTS system can also be seen around the carbon atoms of the benzene ring in PTS (Figure 4c), which is absent in TBS (Figure 4f). These observations point toward the significant interaction of K^+^ with benzene ring carbon atoms in PTS, which can also be correlated to RDFs shown in Figure 1d. The absence of the K^+^ isosurface near the benzene ring in TBS (Figure 4f) agrees well with the RDF depicted in Figure 1f, suggesting low interactions of K^+^ with benzene ring carbons present in TBS. The isosurfaces depicting H_WAT_ are found in the vicinity of oxygen atoms of the sulfonate molecules in PTS and TBS, followed by SDFs representing the O_WAT_. The isosurfaces of O_WAT_ are also forming a circular ring around the carbon atoms of the benzene ring in PTS/TBS. These observations are in agreement with the previous RDF (Figures S5–S7) and *g*(*r*, θ) (Figure 3).

**Figure 4:**
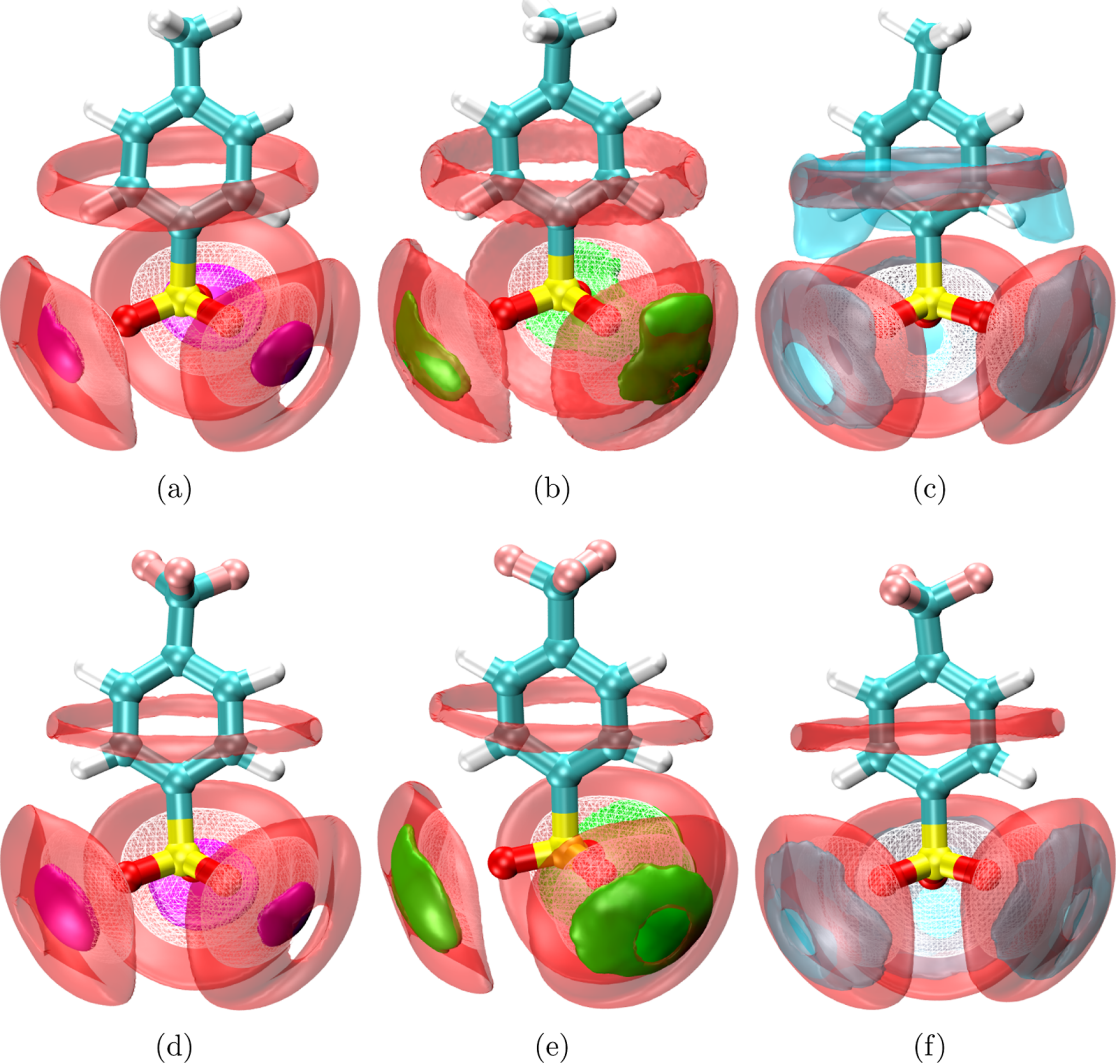
SDFs depicting Li^+^ (magenta), Na^+^ (green), K^+^ (transparent cyan), O_WAT_ (transparent red), and H_WAT_ (white wireframe) around the reference PTS/TBS molecule in (a) Li-PTS, (b) Na-PTS, (c) K-PTS, (d) Li-TBS, (e) Na-TBS, and (f) K-TBS systems. For reference molecules, the hydrogen, carbon, oxygen, sulfur, and fluorine atoms are rendered in white, cyan, red, yellow, and light pink, respectively.

#### 3.2.4 Effect of Solvation

The difference in the interaction of Li^+^ and K^+^ with PTS in water is mainly due to the effect of solvation; Li^+^ is solvated by ∼4 oxygens of water in the first solvation shell and K^+^ is solvated by ∼6 oxygens of water. Figure 5a,b depicts the snapshot of water molecules in the first solvation shell of Li^+^ and K^+^ in the vicinity of the PTS molecule for Li-PTS and K-PTS systems. The higher solvation of K^+^ leads to steric hindrance when it approaches the bulky sulfonate group, and K^+^ has a higher correlation with the planar carbon rings. However, Li^+^ owing to a smaller hydration shell can approach the sulfonate group more easily than K^+^. Therefore, Li^+^ binds to the sulfonate group, while K^+^ binds to the carbon atoms present in the benzene ring of the PTS molecule. However, we observe decreased interactions of K^+^ with carbons present in the benzene ring in TBS because of increased steric hindrance on the benzene ring when hydrogens in the methyl group in PTS are substituted with relatively heavy fluorine atoms in the case of TBS.

**Figure 5:**
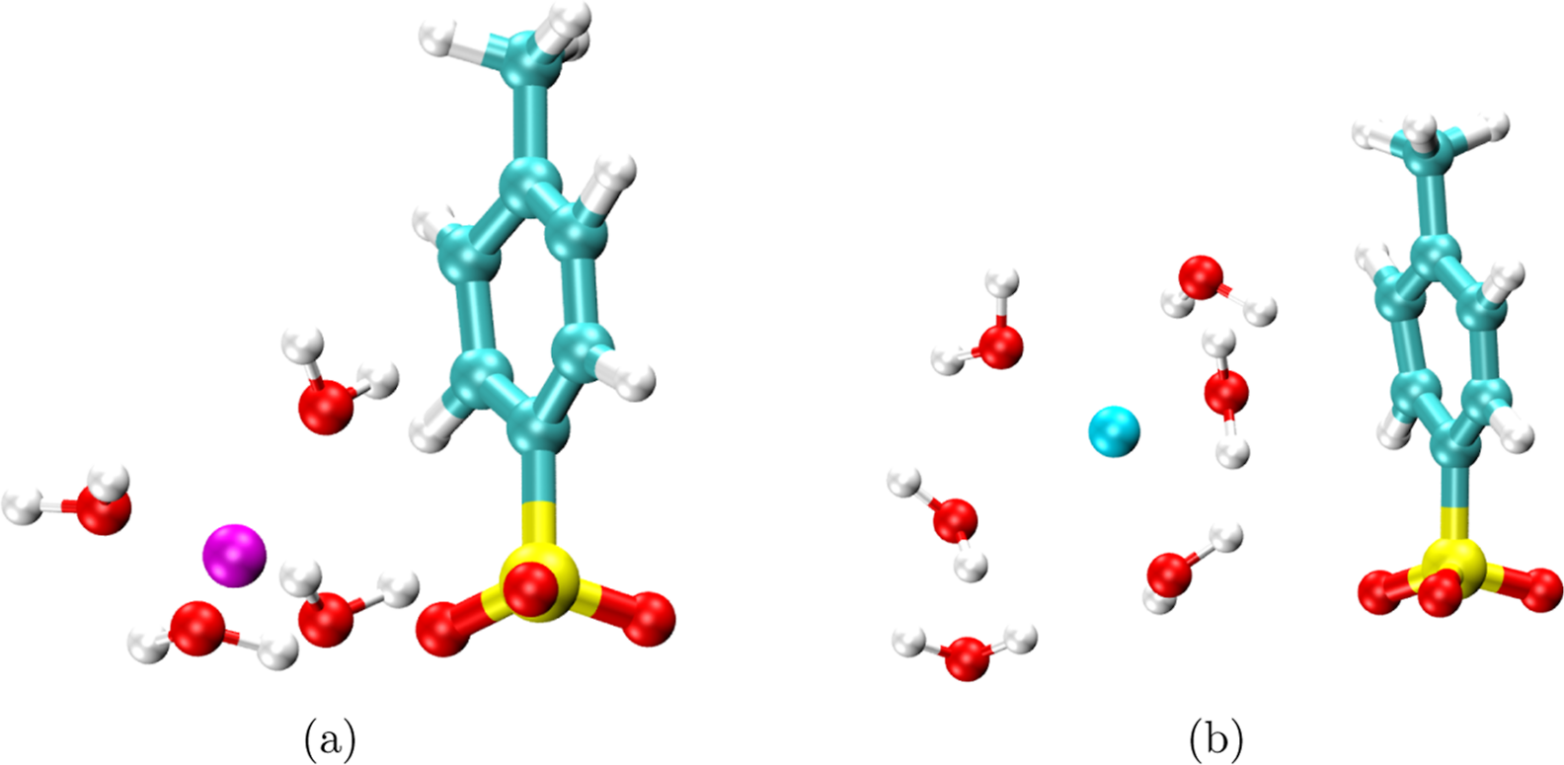
Simulation snapshots showing water molecules in the first solvation shell of (a) Li^+^ and (b) K^+^ in the vicinity of the PTS molecule for Li-PTS and K-PTS systems. The magenta and cyan spheres represent Li^+^ and K^+^, respectively.

#### 3.2.5 Potentials of Mean Force: Comparison of CMD and AIMD Results

Figure 6a,b shows the PMF profiles for cation-PTS and cation-TBS systems along *r* (cation–S_PTS/TBS_) with the closest contact ion-pair (CIP) for Li^+^ at ∼0.33 nm, followed by Na^+^ at ∼0.37 nm, and then K^+^ at ∼0.39 nm, suggesting a close association of Li^+^ with PTS/TBS. The calculated CIP positions agree well with PMFs obtained from both CMD and AIMD simulations. A well-defined barrier separates the CIP and solvent-shared ion-pair (SIP) states, which has water between cations and PTS/TBS in the solvation shell. A high dissociation free energy for converting CIP to SIP is desirable for effectively employing PTS/TBS as an extractant. This free energy is higher for Li^+^ compared to Na^+^ and K^+^. Figure S8 shows that the results are insensitive to the system size.

**Figure 6:**
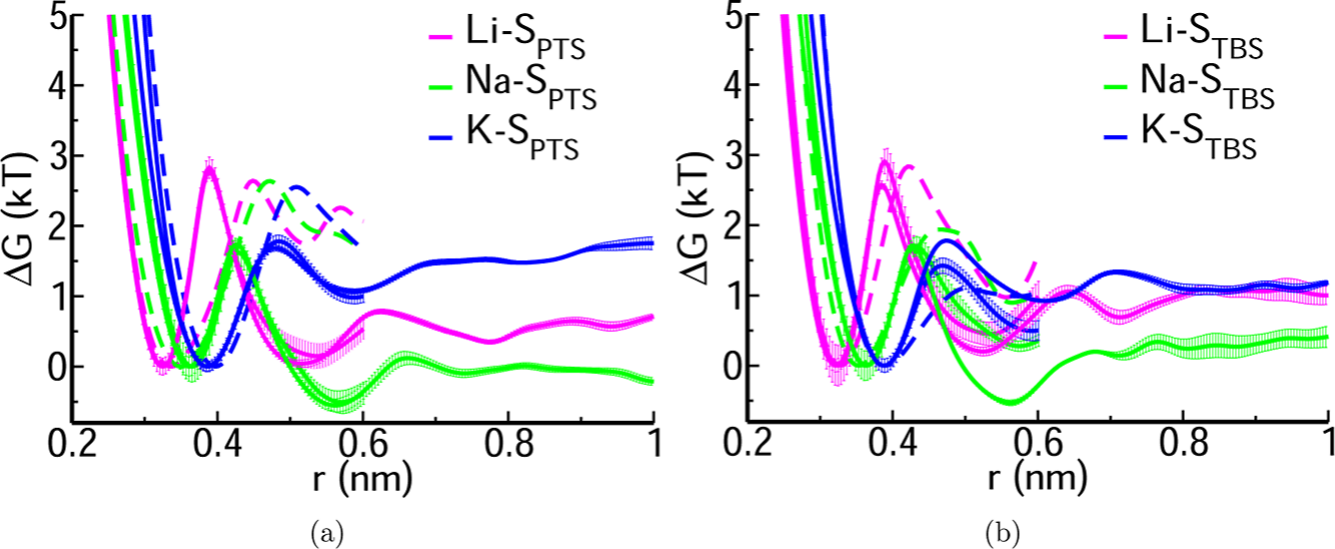
PMF for cation-PTS and cation-TBS in water from CMD (solid lines) and AIMD (dashed lines) simulations along the cation–sulfur distance in the sulfonate group present in (a) PTS (S_PTS_) and (b) TBS (S_TBS_). The PMFs for the smaller system with 64 water molecules are truncated at 0.6 nm, and PMFs for the bigger box size with 1024 water molecules are shown up to 1 nm.

In all cases, however, the minimum for the SIP and the barrier between the SIP and CIP are of order of the thermal energy (∼*kT*). The simulations therefore rule out a strong binding of cations to the sulfonate group and the possibility of long-lived dipoles in solution.

There are notable differences between the PMF profiles obtained from CMD and AIMD simulations, which may be attributed to charge transfer and polarization effects in the AIMD simulations,^64^ which are absent in the CMD simulations, Therefore, quantum treatment is crucial in understanding these systems, and employing polarizable force field models can give more precise results. Figure 6 shows that PMFs calculated for the larger CMD system (1024 water molecules) agree well with the PMFs of the smaller CMD and AIMD systems (64 water molecules), which are truncated at 0.6 nm. At larger distances, the PMF becomes flat, highlighting that the present system is sufficient for a quantitative understanding of the binding free energy.

## 4 Conclusions

We study the interaction between cations (Li^+^, Na^+^, and K^+^) and solutes (PTS, Trimer of PTS, and TBS) in water using classical and AIMD simulations. In the gas phase, the sulfonate group is a binding site for all cations. There is good agreement between DFT and classical force field calculations.

In solution, however, the Li^+^ and Na^+^ ions bind to the sulfonate group but, in the case of PTS, the K^+^ prefers the vicinity of carbon atoms in the benzene ring. We attribute this to the higher CN (6) of water molecules with the K^+^ cation, which makes binding to the planar ring favorable. Interestingly, the interaction of K^+^ with ring carbon atoms decreases for TBS because the hydrogens in the methyl group of PTS are now replaced with the bulky fluorine group in TBS. In addition, the presence of all three cations in solution (with Cl^–^ ions added for electroneutrality) increases the selective binding of Li^+^ to the anion. The binding of Na^+^ is also enhanced when the anion is trimerized.

The free energy of binding of the cations with PTS is weak, only on the order of the thermal energy. It is therefore unlikely that these cations can form long-lived dipole moments with the PTS in solution. The classical force-field simulations are in qualitative but not quantitative agreement with the AIMD simulations, suggesting that polarization and charge transfer are important aspects to consider in these systems.

In conclusion, we find that anions do show selectivity for Li^+^, but the binding is quite weak. Polymerizing the PTS group could lead to enhanced binding and selectivity, and pursuing such a multivalent approach is an interesting direction of investigation.

## Supplementary Material



## Data Availability

The data that support the findings of this study are available from the corresponding author upon reasonable request.

## Notes

Simulation snapshots, RDFs, and finite size results (PDF)
