# Supplementary material for: The interaction of Cations with Solutes in an Aqueous Solution
Source: J Phys Chem B. 2025 Jul 11;129(29):7584–92. doi: 10.1021/acs.jpcb.5c02947 (PMC12503379; doi:10.1021/acs.jpcb.5c02947)
Supplement: Supplementary file 1 [file jp5c02947_si_001.pdf]

Supporting information for The interaction of cations with solutes in aqueous solution

Akshay Malik and Arun Yethiraj*

Department of Chemistry, University of Wisconsin-Madison, Madison, Wisconsin 53706, USA

E-mail: yethiraj@wisc.edu

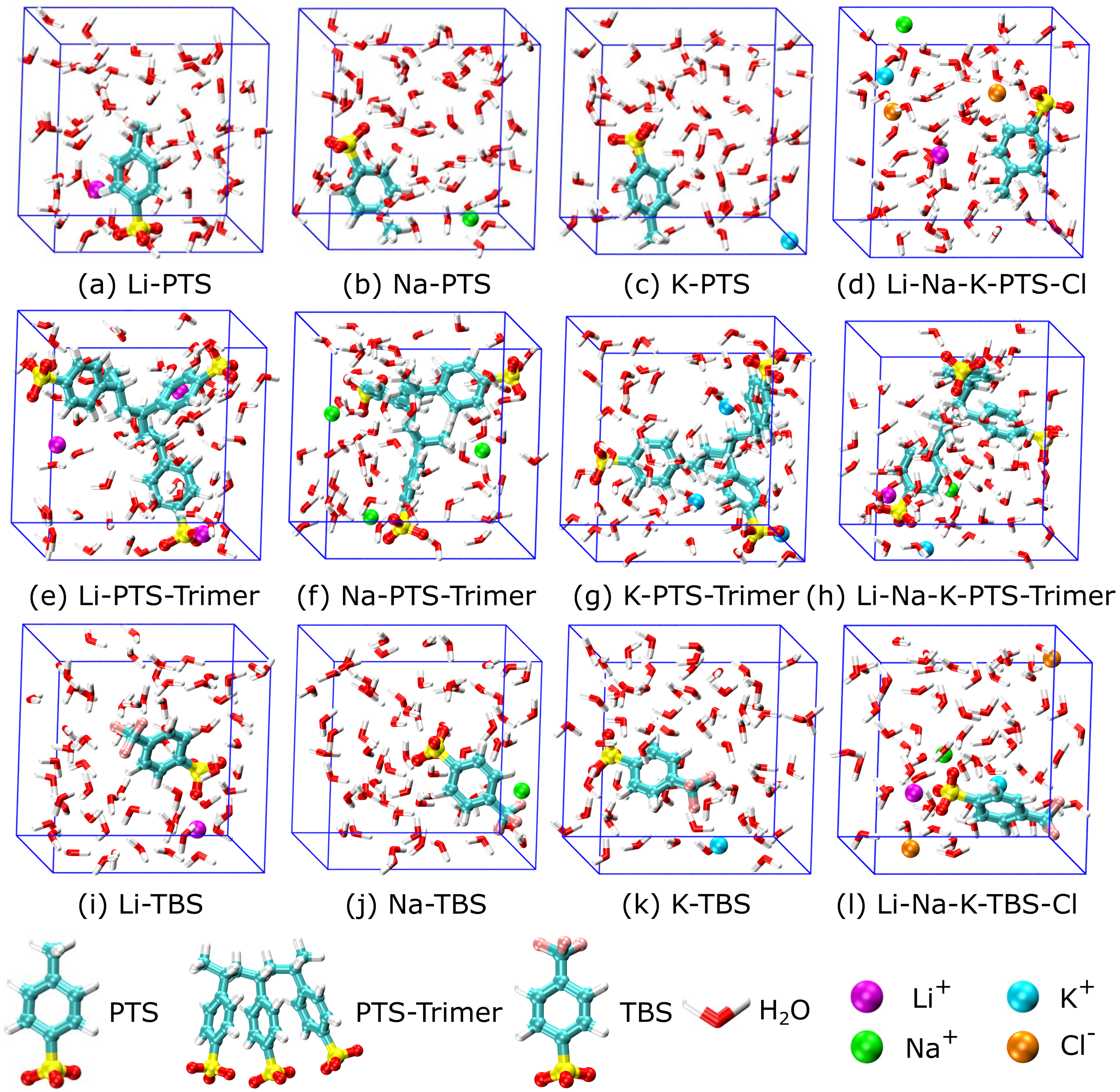

Figure S1: Equilibrated simulation boxes having 64 water molecules and para-toluene sulfonate (PTS)/Trimer of PTS/4-(trifluoromethyl) benzene sulfonate (TBS) mixed with Lithium (Li^+)/Sodium (Na^+)/Potassium (K^+) for different systems (a) Li-PTS, (b) Na-PTS, (c) K-PTS, (d) Li-Na-K with Chloride (Cl^-) ions mixed in a single box (Li-Na-K-PTS-Cl), (e) Li-PTS-Trimer, (f) Na-PTS-Trimer, (g) K-PTS-Trimer, (h) Li-Na-K-PTS-Trimer, (i) Li-TBS, (j) Na-TBS, (k) K-TBS, and (l) Li-Na-K-TBS-Cl. The structures of all the species involved are shown using a ball and stick model.

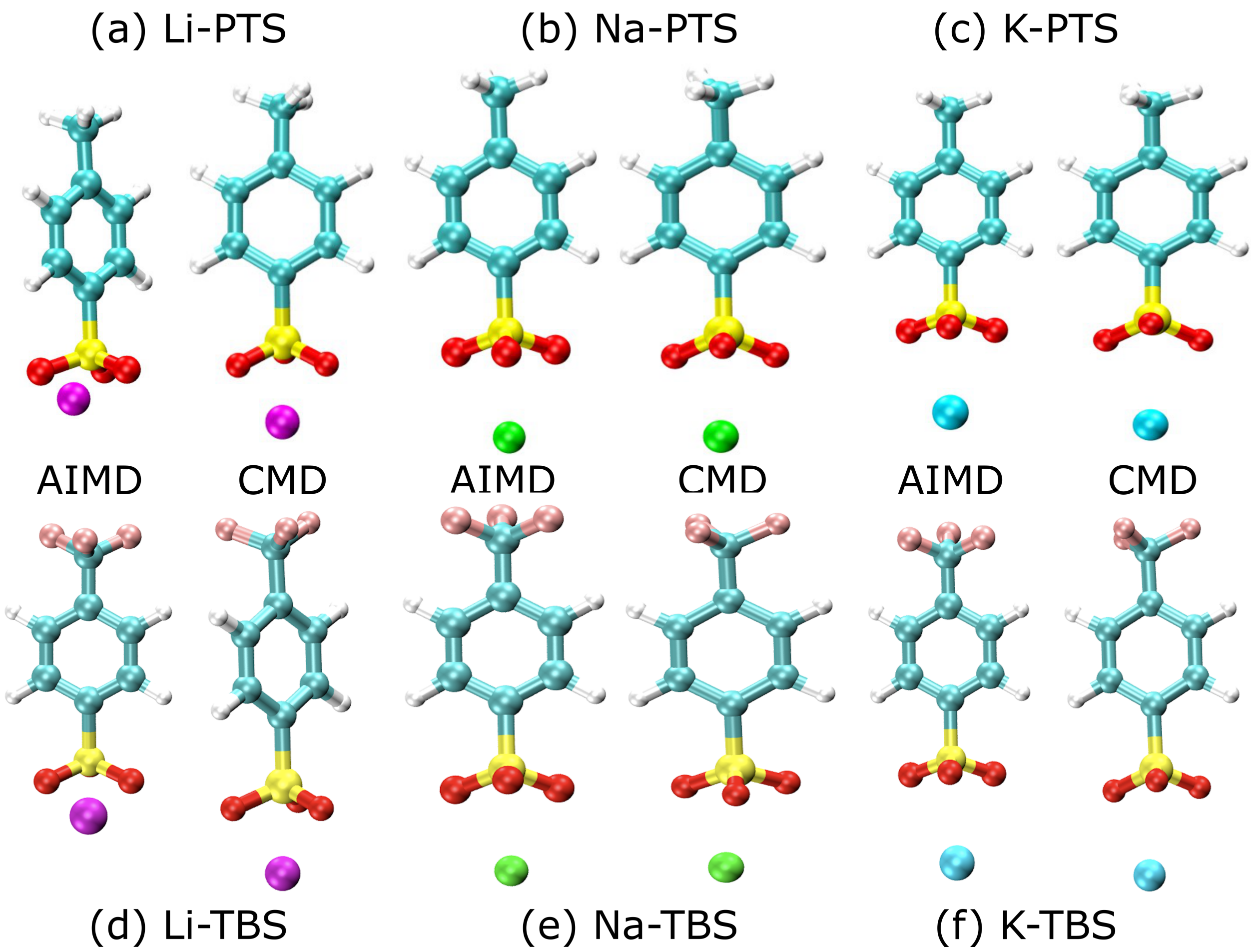

Figure S2: Geometry optimized structures obtained from *ab initio* molecular dynamics (AIMD) simulations and corresponding energy minimized structures obtained from classical molecular dynamics (CMD) simulations in gas phase for systems having (a,d) lithium (Li), (b,e) sodium (Na), and (c,f) potassium (K) with para-toluene sulfonate (PTS) and 4-(trifluoromethyl) benzene sulfonate (TBS), respectively. Here, magenta, green, and cyan spheres represent Li, Na, and K respectively. For PTS and TBS hydrogen, carbon, oxygen, sulfur, and fluorine atoms are represented by white, cyan, red, yellow, and light pink spheres, respectively.

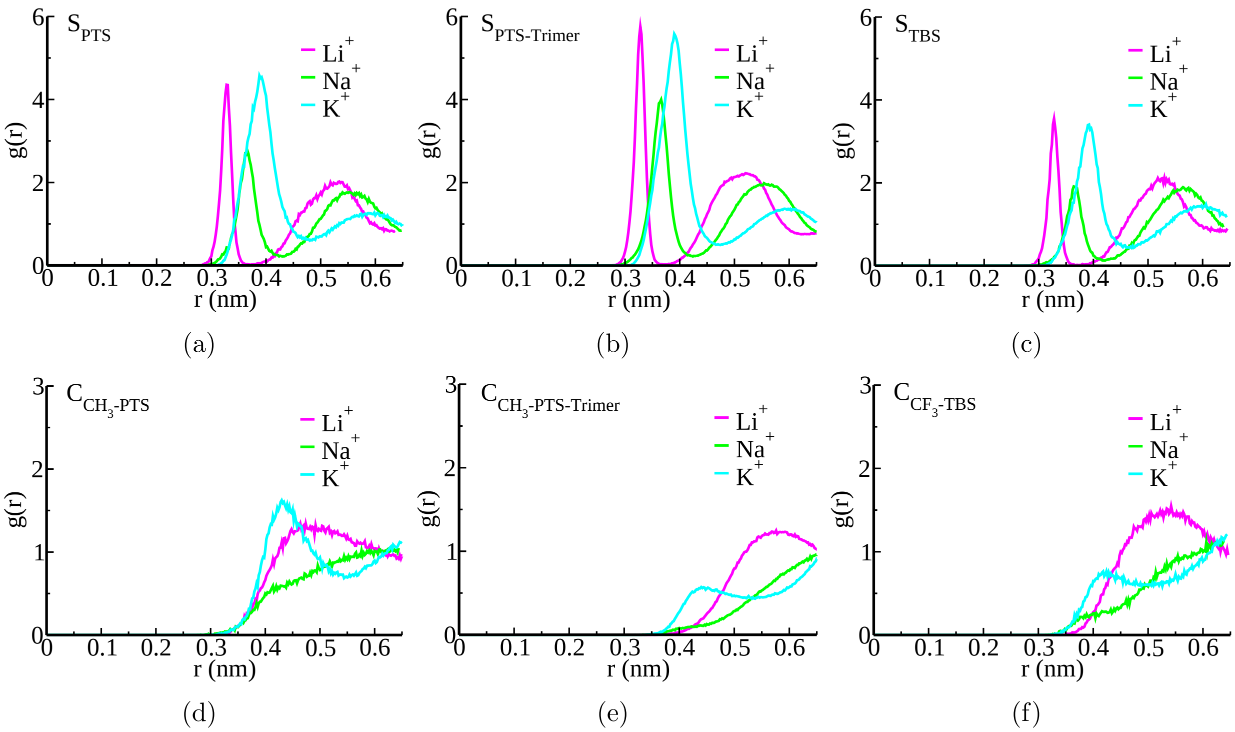

Figure S3: Radial distribution functions for correlations of Li^+, Na^+, and K^+ with sulfur atom in (a) PTS (S_{PTS}), (b) trimer of PTS ($S_{PTS-Trimer}$), (c) TBS (S_{TBS}), and carbon atom directly attached to benzene group present in (d) PTS (C_{CH_3-PTS}), (e) trimer of PTS ($C_{CH_3-PTS-Trimer}$), and (f) TBS (C_{CF_3-TBS}).

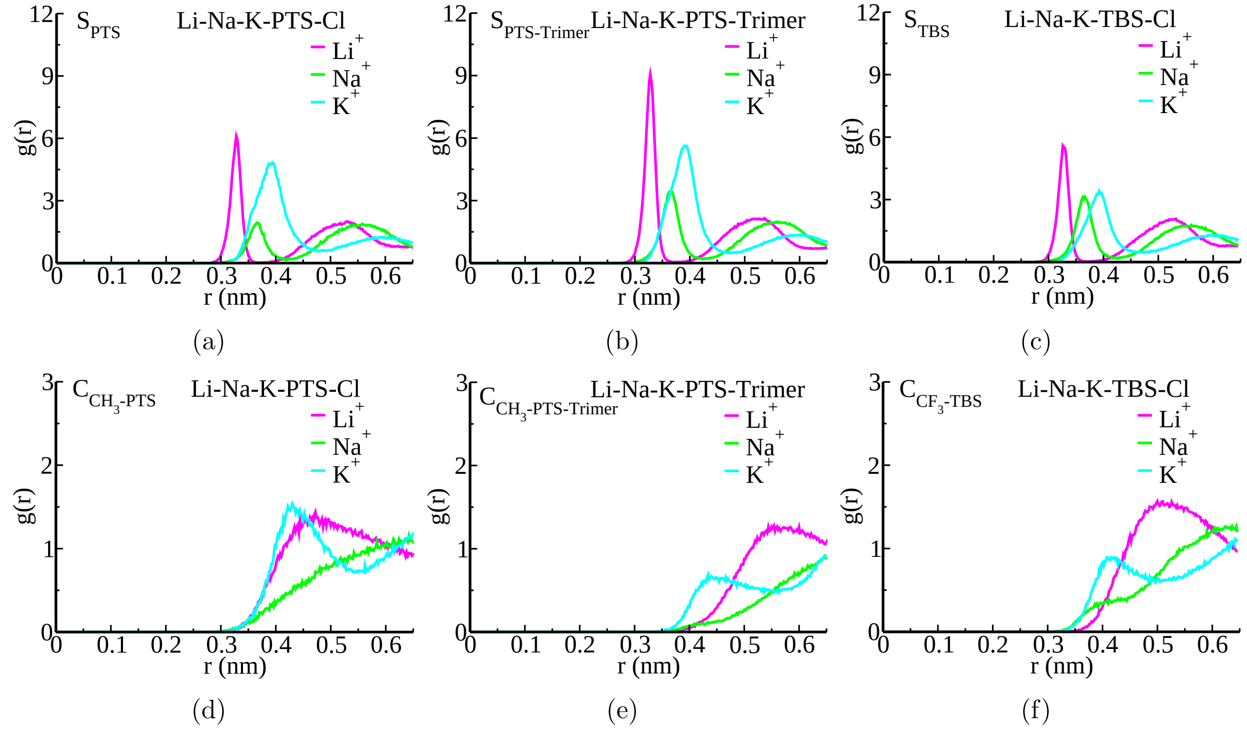

Figure S4: Radial distribution functions for correlations of Li^+, Na^+, and K^+ with sulfur atom in (a) PTS (S_{PTS}), (b) trimer of PTS ($S_{PTS-Trimer}$), (c) TBS (S_{TBS}), and carbon atom directly attached to benzene group present in (d) PTS (C_{CH_3-PTS}), (e) trimer of PTS ($C_{CH_3-PTS-Trimer}$), and (f) TBS (C_{CF_3-TBS}) for a system containing Li^+, Na^+, K^+ mixed with PTS/Trimer of PTS/TBS and Cl^- for Li-Na-K-PTS-Cl, Li-Na-K-PTS-Trimer, and Li-Na-K-TBS-Cl systems solvated in water.

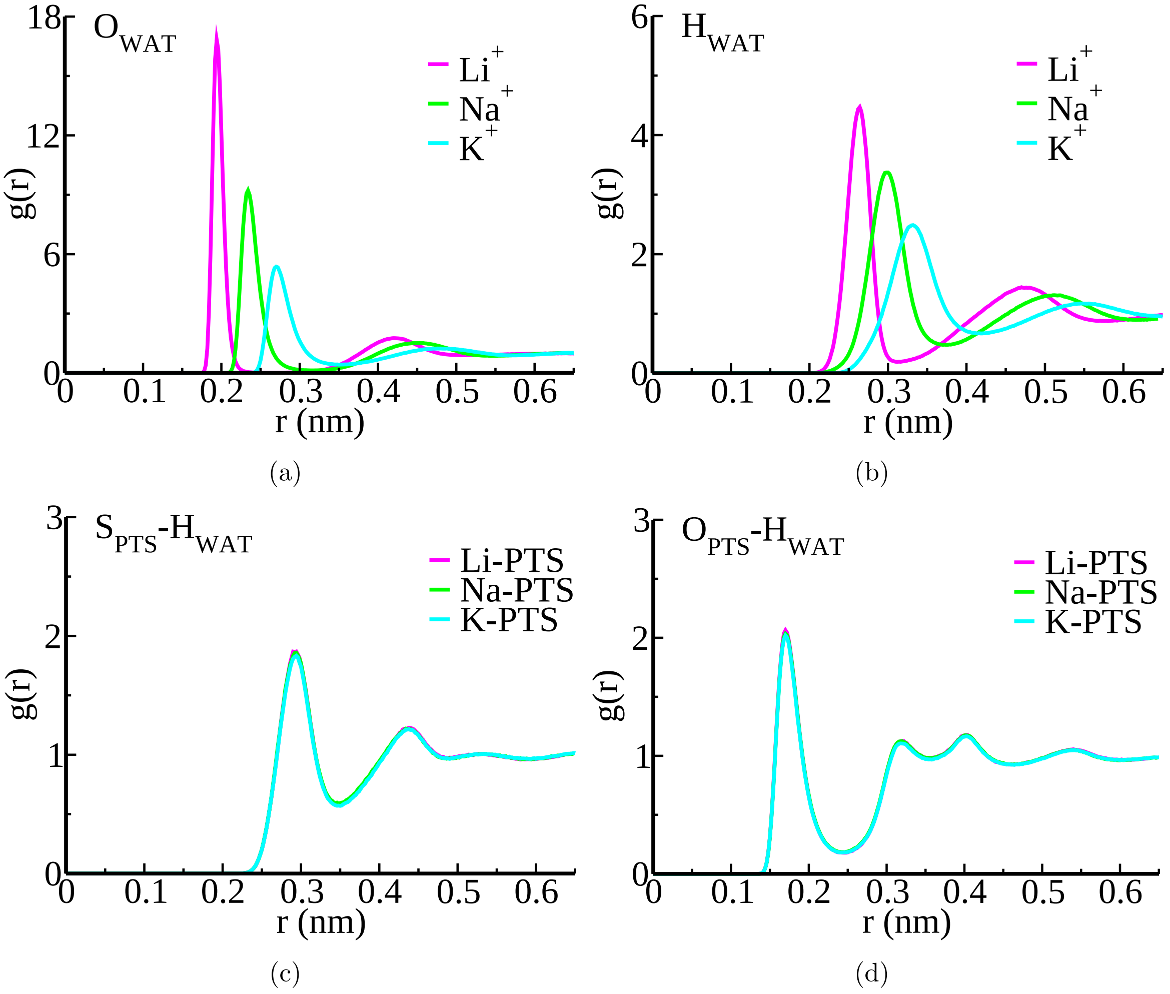

Figure S5: Radial distribution functions for correlations of (a) oxygen (O_{WAT}), (b) hydrogens (H_{WAT}) of water with Li^+, Na^+, and K^+, (c) sulfur atom (S_{PTS}), and (d) oxygen atoms (O_{PTS}) of sulfonate group present in the PTS molecule with H_{WAT} for Li-PTS, Na-PTS, and K-PTS systems.

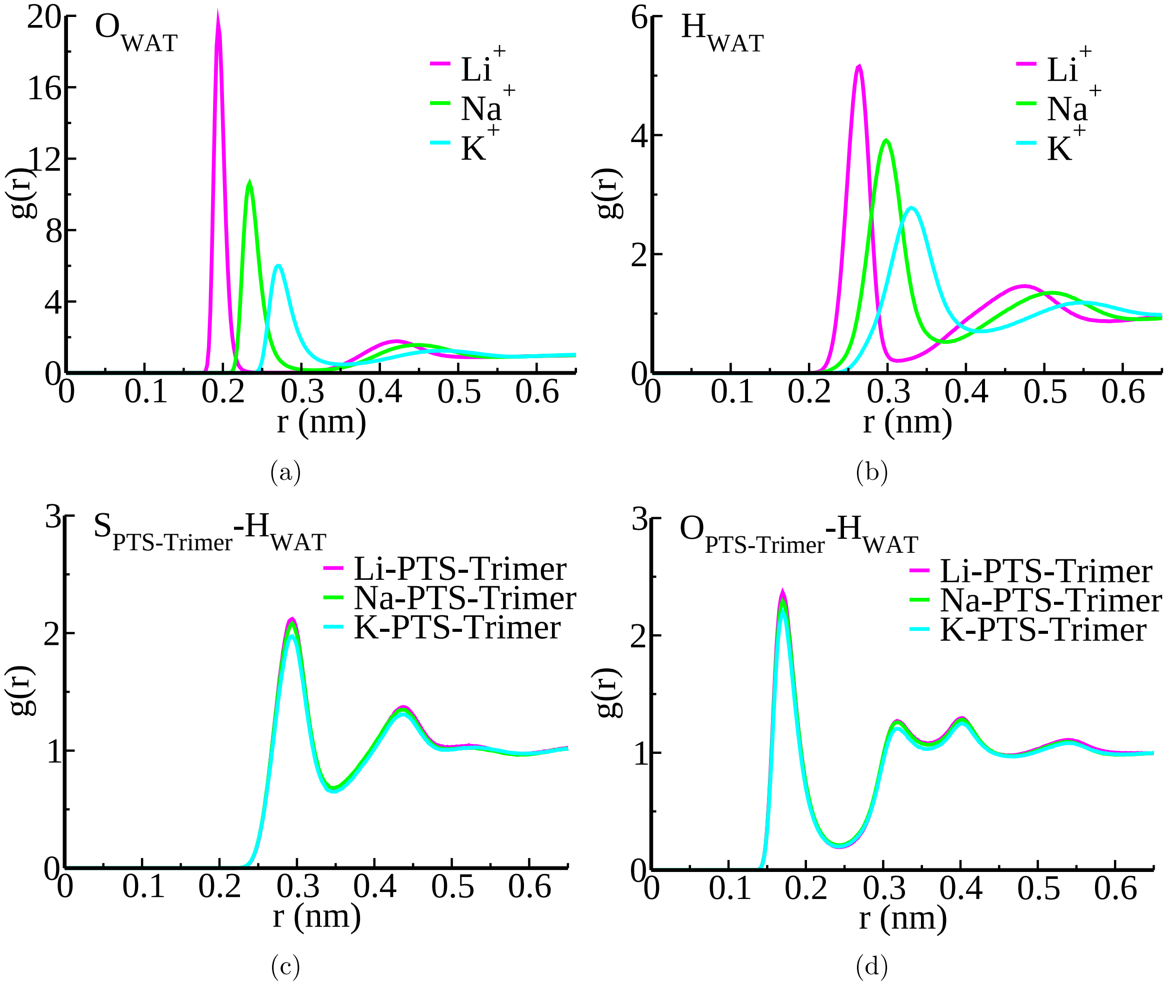

Figure S6: Radial distribution functions for correlations of (a) oxygen (O_{WAT}), (b) hydrogens (H_{WAT}) of water with Li^{+}, Na^{+}, and K^{+}, (c) sulfur atom ($S_{PTS-Trimer}$), and (d) oxygen atoms ($O_{PTS-Trimer}$) of sulfonate group present in the Trimer of PTS molecule with H_{WAT} for Li-PTS-Trimer, Na-PTS-Trimer, and K-PTS-Trimer systems.

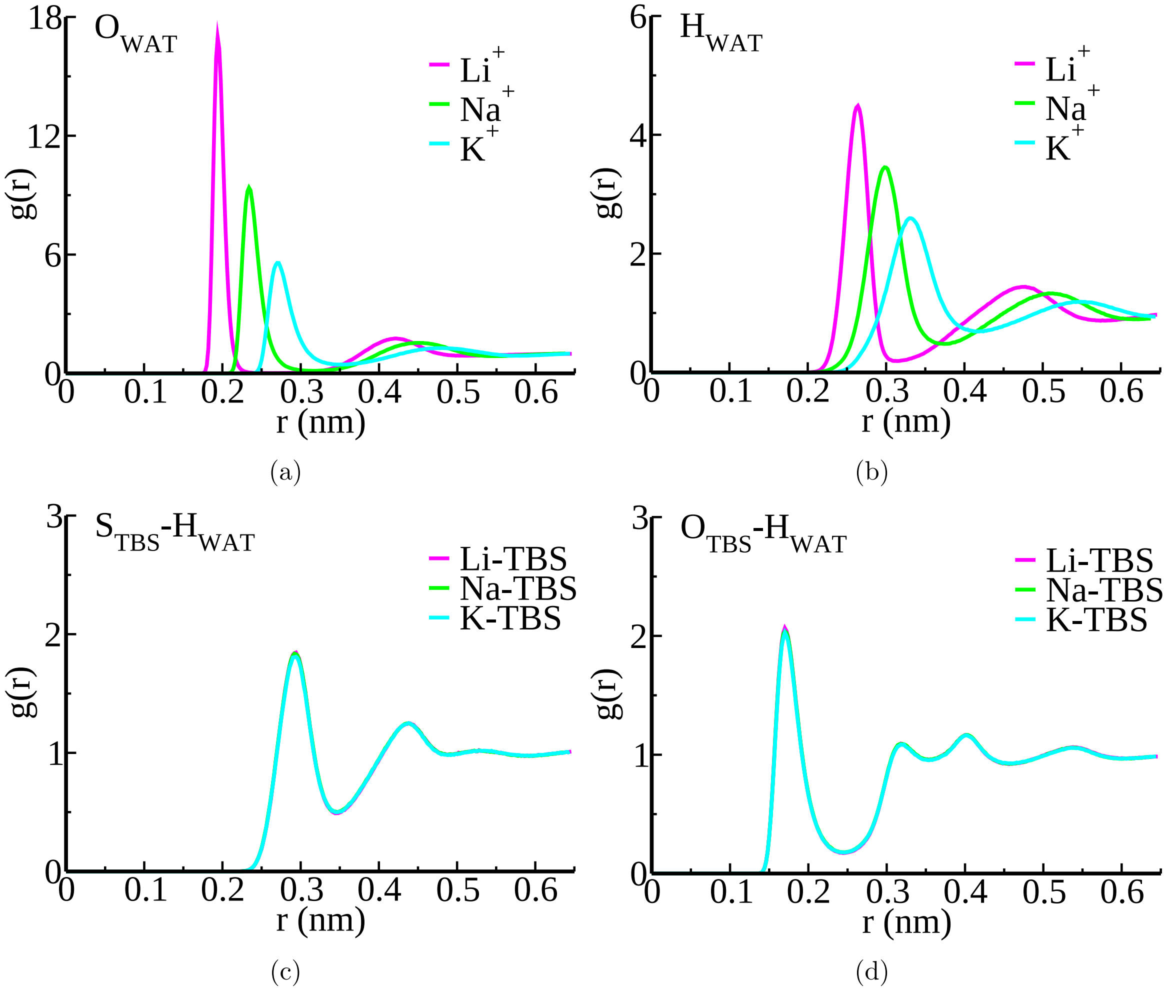

Figure S7: Radial distribution functions for correlations of (a) oxygen (O_{WAT}), (b) hydrogens (H_{WAT}) of water with Li^+, Na^+, and K^+, (c) sulfur atom (S_{TBS}), and (d) oxygen atoms (O_{TBS}) of sulfonate group present in the TBS molecule with H_{WAT} for Li-TBS, Na-TBS, and K-TBS systems.

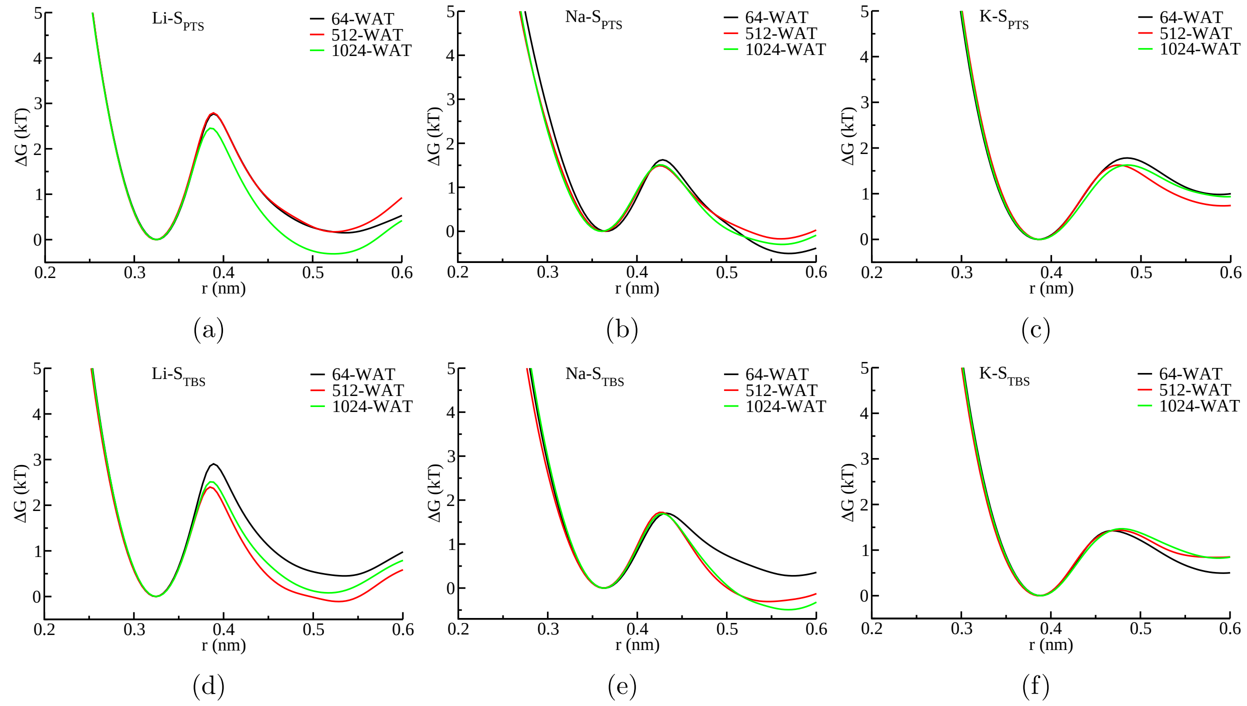

Figure S8: Potential of mean force (PMF) for cation-PTS and cation-TBS in 64, 512, and 1024 water molecules from CMD simulations along the cation-sulfur distance in the sulfonate group present in PTS (a-c) and TBS (d-f).
